# Rivaroxaban in the treatment of livedoid vasculopathy: A long-term retrospective study

**DOI:** 10.1016/j.jdin.2023.10.005

**Published:** 2023-10-30

**Authors:** Sihan Deng, Yu Liu, Jundong Huang, Wei Shi

**Affiliations:** a Department of Dermatology, Xiangya Hospital, Central South University, Changsha, China; b National Clinical Research Center for Geriatric Disorders, Xiangya Hospital, Central South University, Changsha, China

**Keywords:** anticoagulant, coagulopathy, livedoid vasculopathy, rivaroxaban, vasculopathy

*To the Editor*

Livedoid vasculopathy (LV) is a rare thrombotic cutaneous disorder characterized by erythema, painful ulcerations, and atrophie blanche, typically affecting the distal lower portion of the leg. With a recurrent or persistent course, relapse after remission is frequently seen in LV, whereas current approach to induce remission and maintenance remains scarce.^1^ As an oral anticoagulant, rivaroxaban may be preferentially reinstated when symptoms exacerbate due to its convenience and no requirement for monitoring clotting function.^2^^,^^3^ Here, we report a retrospective series using rivaroxaban to assess retreatment and long-term outcomes in patients with LV.

This retrospective study included 34 patients with histologically confirmed LV treated with rivaroxaban at Xiangya Hospital of Central South University between June 2018 to June 2023. The average follow-up duration ± SD was 32.79 ± 13.1 months. All patients received 10 mg of rivaroxaban daily initially, with some adjusting their dosage to either 10 mg every other day or 15 mg every day based on disease severity (Fig 1). Composite clinical scores were used to assess the severity of LV at baseline and recurrence.^4^^,^^5^ The median composite clinical score was 7.0 (IQR: 6.0-9.0) at baseline (Table I). A total of 67.6% (23/34) patients achieved 50% improvement after 2 months of treatment (Supplementary Table I and Supplementary Fig 1, available via Mendeley at https://data.mendeley.com/datasets/xzjsxxk56t/1). Five patients continued to take the drug until the end of follow-up, and the median duration was 33.0 (range: 18.0-38.0) months. Three patients discontinued treatment due to insufficient clinical improvement to meet their expectations (patient 32-34), whereas most patients (26/34) discontinued the medication due to symptom relief. The median preinterruption duration of rivaroxaban was 9.0 (IQR: 3.0-15.5) months. After discontinuation, 10 patients did not experience any recurrence during a median follow-up period of 17.5 (IQR: 7.5-34.75) months, 2 of them did not experience a recurrence for over 3 years. Sixteen individuals had relapsed after discontinuation, and the median time to the first relapse was 7.0 (IQR: 5.0-12) months, with a median severity of 2.5 (IQR: 1.0-3.75). Fourteen patients resumed medication and 12 withdrew again due to disease remission, and the median duration of the second therapy is 3.0 (IQR: 2.0-5.0) months. Overall, 2 patients reported hematochezia, and 6 patients experienced hypermenorrhea. No other adverse reactions were reported.

**Fig 1:**
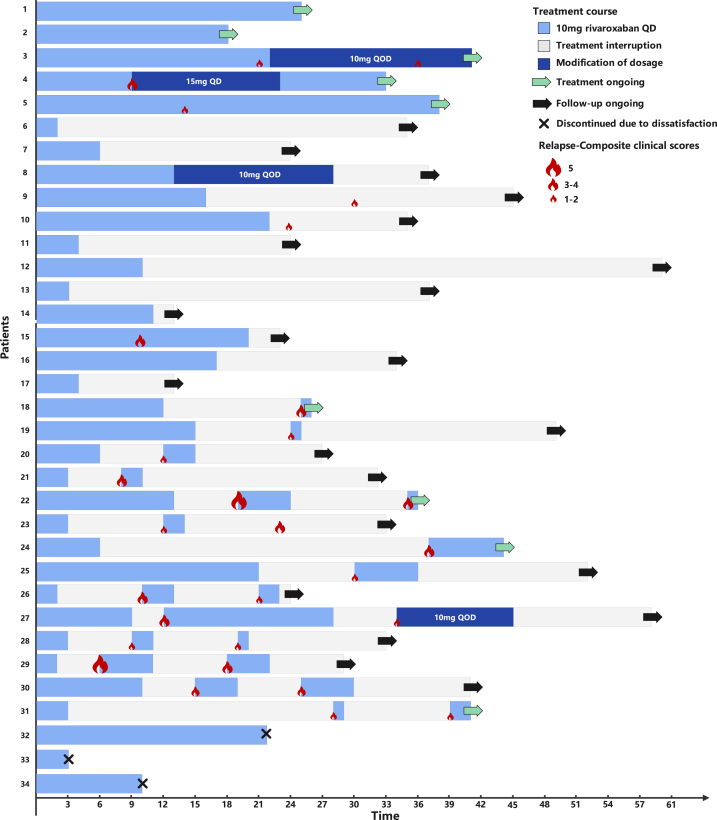
Rivaroxaban treatment course and recurrence of each patient with livedoid vasculopathy.

**Table I:** Characteristics of patients with livedoid vasculopathy

Characteristics	Median (IQR)/number (%)	
Gender		
Female		27 (79.4)
Male		7 (25.9)
Onset age, y		17 (14, 30.25)
Disease’s duration, y		5 (3, 10.25)
Body mass index ± SD		21.14±3.07
Rivaroxaban response time, d		20 (11.5, 21)
	Baseline	2 mo
Total score∗	7 (6, 9)	4 (1.75, 4.25)
Erythema	3 (2, 3)	2 (1, 2)
Ulcer	2 (2, 3)	1 (0-1)
Pain	3 (1.75, 3)	1 (0-1)
Location		
Foot, ankle, and lower portion of the legs		25 (73.5)
Upper portion of the leg		6 (17.6)
Upper extremities		3 (8.8)
Previous medical history		
Atopic dermatitis		7 (20.6)
Hypertension		4 (11.8)
Side effect		
Hematochezia		2 (5.9)
Menorrhagia		6 (17.6)
Immunological examinations†		
Positive antinuclear antibodies		6 (20.7)
Positive antidouble-stranded deoxyribonucleic acid		0 (0.0)
Coagulation studies		
Elevated fibrinogen		4 (11.8)
Decreased antithrombin III		2 (5.9)
Decreased protein C		9 (26.4)
Elevated plateletcrit		21 (61.8)
Elevated C reactive protein		4 (11.8)
Lipoprotein (a) increase‡		4 (12.9)
Homocysteinemia§		7 (23.3)
Concomitant therapy		
Topical mucopolysaccharide polysulfate		15 (44.1)
Colchicine		1 (2.9)
Prednisone		1 (2.9)

∗ Composite clinical scores consists of determination of 3 subscores for erythema, ulceration, and pain. Each subscore ranges from 0 to 3 according to the severity, with 0 indicating normal.

† Data were available except in 5 patients.

‡ Data were available except in 3 patients.

§ Data were available except in 4 patients.

Notably, 3 patients exhibited mild relapses during the summer, they decided against resuming treatment after the relapse and symptoms gradually subsided spontaneously as weather temperature declined. During treatment, 4 patients experienced mild to moderate relapse due to heat exposure and achieved remission after continuing with rivaroxaban. These findings demonstrate that the risk of LV relapse is heightened during the summer and mild to moderate relapses after discontinuation of the medication appear to resolve spontaneously as the temperature drops. Continuous treatment does not guarantee the absence of relapses, but most patients can still achieve remission after retreatment.

This real-world long-term study provides a basis for using rivaroxaban for the maintenance of patients with LV. Long-term use of 10 mg daily rivaroxaban demonstrated positive responses in the Chinese LV population. Individualized dose titration of rivaroxaban, including discontinuation and retreatment based on the severity and preferences may provide a reliable and well-tolerated option to induce remission and maintenance for patients with LV.

## Conflicts of interest

None disclosed.
